# Effects of multidomain lifestyle intervention on frailty among older men and women – a secondary analysis of a randomized clinical trial

**DOI:** 10.1080/07853890.2024.2446699

**Published:** 2025-01-01

**Authors:** Laura Saarela, Jenni Lehtisalo, Tiia Ngandu, Saila Kyrönlahti, Satu Havulinna, Timo Strandberg, Esko Levälahti, Riitta Antikainen, Hilkka Soininen, Jaakko Tuomilehto, Tiina Laatikainen, Miia Kivipelto, Jenni Kulmala

**Affiliations:** a Department of Healthcare and Social Welfare, Services Unit, Finnish Institute for Health and Welfare (THL), Helsinki, Finland; b Department of Public Health, University of Helsinki, Helsinki, Finland; c Department of Public Health, Lifestyles and Living Environments Unit, Finnish Institute for Health and Welfare (THL), Helsinki, Finland; d Institute of Clinical Medicine/Neurology, University of Eastern Finland, Kuopio, Finland; e Division of Clinical Geriatrics, Center for Alzheimer Research, Care Sciences and Society (NVS), Karolinska Institutet, Stockholm, Sweden; f Faculty of Social Sciences (Health Sciences) and Gerontology Research Center (GEREC), Tampere University, Tampere, Finland; g Center for Life Course Health Research, University of Oulu, Oulu, Finland; h University of Helsinki and Helsinki University Hospital, Helsinki, Finland; i Medical Research Center Oulu, Oulu University Hospital, Oulu, Finland; j Neurocenter, Department of Neurology, Kuopio University Hospital, Kuopio, Finland; k National School of Public Health, Madrid, Spain; l South Ostrobothnia Central Hospital, Seinäjoki, Finland; m Institute of Public Health and Clinical Nutrition, University of Eastern Finland, Kuopio, Finland; n Neuroepidemiology and Ageing Research Unit, School of Public Health, Imperial College London, London, UK; o Theme Inflammation and Aging, Karolinska University Hospital, Stockholm, Sweden

**Keywords:** Frailty, healthy aging, RCT

## Abstract

**Background:**

Frailty is a common geriatric syndrome associated with poor clinical outcomes. Effectiveness of lifestyle intervention programmes among frail older people has been examined earlier, but effects of interventions on prevention of frailty have been rarely studied. The aim of this study was to investigate to what extent the multidomain lifestyle intervention in the Finnish Geriatric Intervention Study to Prevent Cognitive Impairment and Disability (FINGER) affected changes in frailty status among older men and women at risk of cognitive disorders.

**Methods:**

The 2-year multidomain lifestyle intervention trial including simultaneous nutritional counseling, physical exercise, cognitive training and social activity, and management of metabolic and vascular risk factors, was conducted among 1259 older people (mean age 68.9 years). A modified Fried’s frailty phenotype (weight loss, exhaustion, weakness, slowness, and low physical activity) was used to assess frailty at baseline and after the 2-year intervention. Participants with one or more components of the frailty phenotype were classified as pre-frail or frail. A multinomial regression model was applied to investigate efficacy of the intervention on frailty.

**Results:**

We observed a favorable trend in reversing frailty among older men with the intervention. Pre-frail or frail men in the intervention group had higher probability of being non-frail after the intervention (44%) than pre-frail or frail men in the control group (30%) (*p* = 0.040). Among men, the intervention was especially beneficial in terms of increasing physical activity. Among women, multidomain lifestyle intervention did not affect the frailty status.

**Conclusion:**

Modifying lifestyle-related factors may have potential to reverse first signs of frailty among older men. However, the intervention lasted only two years, therefore, research with longer follow-up is needed to see possible long-term effects of lifestyle management on the development of frailty.

## Background

Frailty in old age refers to the clinically recognized decline in health and functioning which is not caused by any specific disease. It is associated with the risk of adverse health outcomes such as poor quality of life [1], disability, falls, hospitalization [2–4], long-term care [5], and death [2,4,6,7]. Frailty is a complex condition with a multifactorial origin. A widely used method to assess frailty, the Fried’s phenotype [4], incorporates physical aspects including weight loss, exhaustion, low physical activity, weakness, and slowness.

With the rapidly growing proportion of older adults in many populations, the prevalence of frailty is expected to rise sharply [8]. In addition to effects on personal daily life and functioning of individual older persons, this will result in significant health and societal expenses. A recent study found that the mean social care costs for persons with frailty living in their own homes were approximately nine times higher than for their non-frail counterparts [9]. Preventing and reversing frailty is therefore essential at both the individual and societal levels.

Several of risk factors of frailty, such as low physical activity and anorexia of aging, are potentially preventable or reversible providing opportunities for effective interventions [8]. While some intervention studies have indicated that lifestyle interventions may reduce frailty among frail or pre-frail community-dwelling older people [10], clinical trials including also non-frail persons and addressing both primary and secondary prevention of frailty are lacking.

The efficacy of single and multidomain interventions to treat frailty and prevent associated adverse outcomes in older people has been investigated previously [11–13]. A review [13] suggested that multidomain interventions are likely more effective than single domain interventions in impacting frailty status and its severity. Positive impact of exercise programs among frail older individuals has been demonstrated in various studies, and physical activity plays a pivotal role in multidomain interventions [14–16]. A multinational multicomponent intervention based on physical activity and nutritional counseling in older adults with physical frailty and sarcopenia was associated with a reduction in the incidence of mobility disability [17]. Combinations of exercise and nutritional supplementation have also proven successful in preventing frailty [11,18]. Additionally, a multidomain intervention encompassing cognitive training, nutritional counseling, advice on physical activity, and physician consultations to manage cardiovascular risk factors was associated with a reduced risk of developing frailty among community-dwelling older adults, although without impact on the severity of frailty [19]. Notably, this study was among the few [19,20] that had a relatively long intervention duration, exceeding one year, unlike most large multidomain randomized controlled trials (RCT). In addition, there are differences in risk factors for frailty among men and women [21] and therefore, also intervention effects may vary between the sexes.

We hypothesized that the intervention used in the Finnish Geriatric Intervention Study to Prevent Cognitive Impairment and Disability (FINGER) that was previously found beneficial for cognition [22] and physical functioning [23] may also influence the frailty status among the FINGER participants. Therefore, the aim of this study was to investigate the efficacy of a 2-year multidomain lifestyle intervention in preventing and reversing frailty among relatively healthy older men and women at risk of cognitive disorders.

## Methods

### Participants

**Figure 1.**
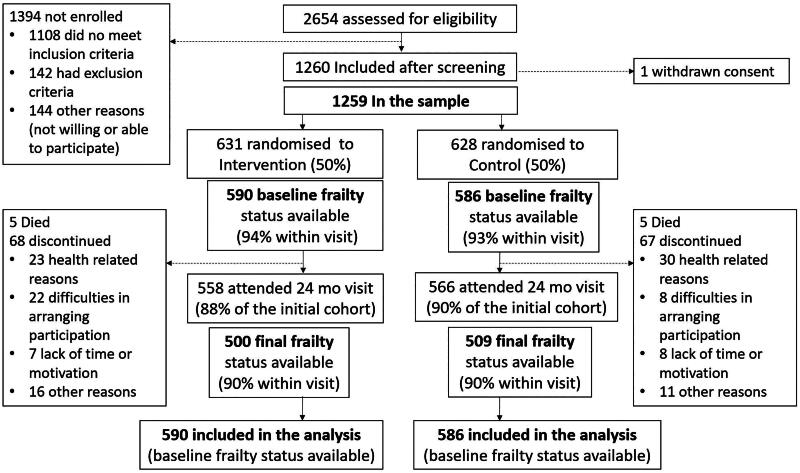
Flowchart of the study.

The study population consisted of independently living individuals, who participated in the FINGER study [24]. The study was carried out at six centers in Finland: Helsinki, Kuopio, Oulu, Seinäjoki, Turku, Vantaa, with 1259 individuals aged 60–77 years at baseline. The flowchart of the study is depicted in Figure 1. The participants were randomized into the intervention (*n* = 631, 45% women)) and control (*n* = 629, 48% women) groups. All participants had the Cardiovascular Risk Factors, Aging and Dementia (CAIDE) risk score [25] of ≥ six points, and cognitive performance at the mean level or slightly lower than expected for their age in the Finnish population, tested with Consortium to Establish a Registry for Alzheimer’s Disease (CERAD) neuropsychological test battery [26]. Individuals with substantial cognitive impairment (based on previously diagnosed dementia, Mini Mental State Examination (MMSE) [27] < 20 points, or clinical judgement), or disorders affecting safe participation in the intervention (e.g. malignant disease, major depression, symptomatic cardiovascular disease, revascularization within one year), severe loss of vision, hearing, or communicative ability, and coincident participation in another intervention trial were excluded from the study. The FINGER study protocol and baseline characteristics are described in detail earlier [24,28] and it is registered in ClinicalTrials.gov database (identifier: NCT01041989). The research followed ethical principles established in the Declaration of Helsinki. The participants gave their written consent before enrollment to the study, and the FINGER study protocol was approved by the ethics committee of the Helsinki and Uusimaa Hospital district (approval number: HUS/1204/2017), which issues all the ethical approvals for studies involving human subjects that are conducted within the district [24]. We have adhered to the CONSORT guidelines in reporting of this study.

### Intervention

Participants were randomly assigned into intervention and control group at a 1:1 ratio and they were not actively informed about the group allocation. Before randomization, both groups received written and oral information and advice on healthy diet and physical, cognitive, and social activities beneficial for management of vascular risk factors and disability prevention. The intervention components have been described in detail earlier [22,24]. Briefly, intervention group received intensive two-year multidomain intervention, which included simultaneous nutritional counseling, physical exercise, cognitive training and social activity, and management of metabolic and vascular risk factors. The nutritional guidance was based on the Finnish Nutrition Recommendations [29], and it included group (7–9 sessions) and individual sessions (3 sessions). The physical exercise was based on international guidelines [30] and represents a modified version of the Dose-Responses to Exercise Training (DR’s EXTRA) study protocol [31], including individually tailored progressive muscle strength training (1–3 times per week) and aerobic exercise (2–5 times per week), and exercises to maintain and improve postural balance. Cognitive training program was adapted for the FINGER from protocols from previous trials [32] and it included group sessions (10 sessions) and individual, independent computer-based training (144 sessions in total). The social activities were stimulated through the numerous group meetings in all the intervention components. The monitoring and maintenance of metabolic and vascular factors were based on national evidence-based guidelines [33–35].

### Definition of frailty

The frailty status was determined using the modified Fried’s phenotype [4], which consists of five frailty components (weight loss, exhaustion, low physical activity, weakness and slowness) comprising a score ranging from 0 to 5. Higher score indicates the presence of more frailty components. Detailed definitions of the components are shown in Table 1. All the components except for weight loss, were assessed similarly at baseline and at 2-year follow-up. *Weight loss:* Participants who during the previous year had lost 5% or more of their body weight or over 4.5 kg were considered as having weight loss. At baseline and after 12 months it was assessed with a self-reported question: *How much does your current weight differ from your previous year weight?.* Weight loss at 24 months follow-up was calculated from self-reported weight at 12 months and 24 months follow-up visits. *Exhaustion* was assessed with a question about weakness or tiredness over the previous month. Participants reporting quite a lot or very much weakness or tiredness were considered as having exhaustion. *Low physical activity* was assessed with two questions: *How often do you participate in leisure-time physical activity that lasts at least 20 min and causes breathlessness and sweating?* and *How many minutes per day on average do you take up other leisure time activities which require physical activity?* Participants who reported physical activity once a week or less, and less than 15 min daily were considered as being physically inactive. *Weakness* was determined based on hand-grip strength test, which was measured with a hydraulic hand dynamometer (Saehan SH 500, Saehan Co, Korea). The measurement was performed with the participant sitting with their arm bent at 90-degree flexion by the body. The best of two measures from the dominant hand was used. In case the dominant hand was sore, injured or the measurement was missing, the non-dominant hand measurement was used. If the dominant hand was not known, measurement from the right hand was used. To determine weakness, sex and body mass index-adjusted cutoff points proposed by Fried et al. [4] were used. *Slowness* was defined by adapting the criteria proposed by Fried et al. [4] using the four-metre usual gait speed timed with a hand-held stopwatch. The better of two performances was used, and cutoff points were adjusted for sex and height.

**Table 1.** Definition of frailty.

Weight loss	Weight loss of 5% or more or over 4.5 kg during the previous year were categorized as weight loss.	At baseline self-reported question: ‘How much does your current weight differs from your previous year weight?’ (Weight last year defined using baseline weight and this difference; and percentage of weight loss calculated as the reduced weight divided by weight last year) At 24 m: Weight assessed at the visit; weight loss calculated as difference between 12-month and 24-month weight divided by 12-month weight)			
Exhaustion	Self-reported quite a lot or very much.	Self-reported question: ‘Have you experienced weakness or tiredness over the previous month (30 days)?’ Response options not at all, quite little, some, quite a lot, and very much.			
Low physical activity	Self-reported leisure time physical activity once a week or less, and other leisure-time activities less than 15 min daily.	Two self-reported questions: ‘How often do you participate in leisure-time physical activity that lasts at least 20 min and causes breathlessness and sweating?’ Response options: five times a week or more often, four times a week, three times a week, two times a week, once a week, less than once a week, not at all due to a disease or physical disability; and ‘How many minutes per day on average do you take up other leisure time activities which require physical activity?’ Response options: less than 15 min daily, 15–29 min daily, 30–59 min daily, one hour or more daily. Participants reporting physical activity once a week or less, and less than 15 min daily were considered as being physically inactive.			
Weakness	Grip strength from dominant hand, stratified by gender and body mass index (BMI). Cut-off for grip strength (kg) criterion for frailty.	Men:	Grip strength	Women:	Grip strength
		BMI	(kg)	BMI	(kg)
		≤24.00	≤29	≤23.00	≤17
		24.01–26.00	≤30	23.01–26.00	≤17.3
		26.01–28.00	≤30	26.01–29.00	≤18
		>28.00	≤32	> 29.00	≤21
Slowness	4-Metres usual walking speed, stratified by gender and height. Cut-off for times for frailty.	Height		Walking time	
		Men	Women		
		≤173 cm	≤159 cm	≥6.15 sec	
		>173 cm	>159 cm	≥5.26 sec	
Total		Participants with three or more components were classified as frail, one to two as pre-frail, and none as non-frail. Pre-frail and frail groups were combined due to limited number of frail participants.			

First, participants with frailty score of ≥3 were classified as frail, those with 1–2 as pre-frail, and those with 0 as non-frail [4]. For the final analyses pre-frail and frail participants were combined into one group due to very few participants with frailty. Non-frail group included study participants with no missing data and without any frailty components. Participants belonging to the pre-frail/frail group had at least one component of frailty. This group also included participants who did not have complete data on frailty available, but based on available data were pre-frail or frail. Study participants who had fully missing data on frailty or available data did not enable the frailty status assessment at 2-year follow-up were categorized into third group with no information available. Persons with missing frailty data already at baseline (*n* = 83) were excluded from the analyses.

### Statistical analyses

Baseline differences between frailty groups were tested with *t*-test and *χ*^2^ tests. A multinomial regression model was applied to investigate the effect of the intervention on frailty status, including all participants with baseline frailty status available, regardless of participation in the intervention activities (intention to treat analysis). The outcome was post-intervention frailty status and persons with missing data on follow-up frailty were included as one of the post-intervention groups to account for possible non-randomness of missing data. The analyses were adjusted for study site, sex, baseline sum of chronic diseases (categorized as none, 1, 2, or ≥3), age and years of education. Also, the interactions between intervention group and baseline frailty and sex status were investigated. *Margins* command was used to predict the probabilities of each level of frailty status after 24 months. Differences in these probabilities between intervention and control group were tested with commands *test* and *nlcom* and reported with 95% confidence intervals. *p*-Value of <0.05 was considered statistically significant. All analyses were performed with Stata 18.0 software.

## Results

At baseline, the mean age of the participants was 68.9 (SD 4.7) and 47% were women. Out of 1259 participants, 1176 (93%) had baseline frailty definition available (Figure 1). Of them 358 persons (28%) were pre-frail or frail (including 349 pre-frail and 9 frail persons). Those with pre-frailty or frailty had higher BMI and more chronic diseases than the non-frail group at baseline (Table 2). Those with missing information on frailty at baseline (*n* = 83, 7%) were more often women (59% of those with missing data, *p* = 0.019) but otherwise they were not different from those with available frailty status.

**Table 2.** Baseline characteristics of the FINGER study population by baseline frailty status.^a^

Characteristic	Information available (*n*)	Non-frail (*n* = 818)	Prefrail or frail (*n* = 358)	*p*-Value^c^
Women (*n*, %)	1176	367 (44.9%)	171 (47.8%)	0.358
Age (years)	1176	68.6 (4.6)	69.1 (4.8)	0.104
Education (years)	1175	10.0 (3.5)	9.9 (3.4)	0.680
Body mass index, (kg/m^2^)	1173	27.7 (4.3)	29.5 (5.3)	<0.001
Sum of chronic diseases^b^	1139			<0.001
None, (*n*, %)		167 (21.0%)	34 (9.9%)	.
One (*n*, %)		251 (31.6%)	76 (22.9%)	
Two (*n*, %)		192 (24.2%)	111 (32.3%)	
Three or more (*n*, %)		185 (23.3%)	123 (35.8%)	
Intervention group (*n*, %)	1176	404 (49.4%)	186 (52.0%)	0.418

^a^ Data are given as number (percentage) or mean (SD).

^b^ Sum of self-reported chronic diseases during the later life assessments: high blood pressure, heart failure, angina pectoris, cancer, asthma, pulmonary emphysema or chronic bronchitis, gallstones or gall bladder inflammation, rheumatoid arthritis, other articular disease, back illness, chronic urethritis or nephritis, cerebrovascular disease, diabetes, depression, and other psychological illnesses.

^c^ *p*-Value for difference between groups, chi^2^-test (categorical variables) or *t*-test (continuous variables), as appropriate.

There was no difference in the prevalence of frailty between the intervention and control groups at baseline (Table 3). After the 2-year intervention period, 962 persons had data on frailty available from both baseline and 2-year follow-up (81% of those with baseline data) and 30% from them were pre-frail or frail. Among those without frailty information at 2 years, 81 (38%) were pre-frail or frail at baseline. The most common components of frailty were weight loss, weakness, and exhaustion (Table 3). The study participants (*n* = 214) without frailty information available after the intervention were older (*p* = 0.006) compared with participants with frailty information available at follow-up.

**Table 3.** Prevalence of the frailty components at baseline and after 2-year intervention.

	Baseline status			24 Month status (among those with baseline status available)		
Characteristics	Total (*N* = 1259)	Intervention (*N* = 631)	Control (*N* = 628)	Total (*N* = 1176)	Intervention (*N* = 590)	Control (*N* = 586)
Frailty status	*p* = 0.714^a^			*p* = 0.534^a^		
No frailty	818 (65.0%)	404 (64.0%)	414 (65.9%)	677 (57.6%)	344 (58.3%)	333 (56.8%)
Pre-frail or frail	358 (28.4%)	186 (29.5%)	172 (27.4%)	285 (24.3%)	135 (22.9%)	150 (25.6%)
No information	83 (6.6%)	41 (6.5%)	42 (6.7%)	214 (18.2%)	111 (18.8%)	103 (17.6%)
Weight loss	*p* = 0.469^a^			*p* = 0.361^a^		
No frailty component	1140 (90.6%)	565 (89.5%)	575 (91.6%)	1036 (83.1%)	510 (81.7%)	526 (84.6%)
Frailty component	106 (8.4%)	59 (9.4%)	47 (7.5%)	62 (5.0%)	33 (5.2%)	30 (4.8%)
No information	13 (1.0%)	7 (1.1%)	6 (1.0%)	148 (11.9%)	82 (13.2%)	66 (10.6%)
Exhaustion	*p* = 0.634^a^			*p* = 0.457^a^		
No frailty component	1152 (91.5%)	573 (90.8%)	579 (92.2%)	994 (80.8%)	488 (79.4%)	506 (82.1%)
Frailty component	79 (6.3%)	42 (6.7%)	37 (5.9%)	70 (5.7%)	38 (6.2%)	32 (5.1%)
No information	28 (2.2%)	16 (2.5%)	12 (1.9%)	167 (13.6%)	89 (14.5%)	78 (12.8%)
Low physical activity	*p* = 0.356^a^			*p* = 0.368^a^		
No frailty component	1162 (92.3%)	576 (91.3%)	586 (93.3%)	1028 (83.8%)	511 (83.2%)	517 (84.3%)
Frailty component	65 (5.2%)	38 (6.0%)	27 (4.3%)	40 (3.3%)	17 (2.8%)	23 (3.8%)
No information	32 (2.5%)	17 (2.7%)	15 (2.4%)	159 (13.0%)	86 (14.0%)	73 (11.9%)
Weakness	*p* = 0.845^a^			*p* = 0.710^a^		
No frailty component	1051 (83.5%)	528 (83.7%)	523 (83.3%)	865 (72.1%)	433 (72.2%)	432 (72.0%)
Frailty component	149 (11.8%)	72 (11.4%)	77 (12.3%)	156 (13.0%)	74 (12.3%)	82 (13.7%)
No information	59 (4.7%)	31 (4.9%)	28 (4.5%)	179 (14.9%)	93 (15.5%)	86 (14.3%)
Slowness	*p* = 0.895^a^			*p* = 0.664^a^		
No frailty component	1187 (94.3%)	593 (94.0%)	594 (94.6%)	1013 (83.7%)	501 (82.8%)	512 (83.7%)
Frailty component	23 (1.8%)	12 (1.9%)	11 (1.8%)	24 (2.0%)	12 (2.0%)	12 (2.0%)
No information	49 (3.9%)	26 (4.1%)	23 (3.7%)	173 (14.3%)	92 (15.2%)	81 (13.4%)

^a^ *P*-values from chi^2^ test including frailty status and the study group.

There was no difference between the intervention and control groups in the incidence of frailty among all participants (*p* = 0.381). Neither baseline frailty status (group*baseline frailty interaction *p* = 0.353) nor sex alone modified this effect (group*sex interaction *p* = 0.521). There was, however, a three-way-interaction between the group, baseline frailty status, and sex (*p* = 0.040). At the 2-year follow-up, participants in the intervention group who were pre-frail or frail at baseline had numerically less often pre-frailty or frailty (39%) than those in the control group (49%), but the difference did not reach statistical significance (*p* = 0.059) (Table 4). Among men, those in the intervention group who were pre-frail or frail at the baseline, had a higher probability of being non-frail after 24 months (45%) than pre-frail or frail men in the control group (30%) (*p* = 0.036). Consequently, 45% of men in the control group who were pre-frail or frail at baseline stayed in pre-frail group when the corresponding percentage was 28% in intervention group (*p* = 0.015). Differences in frailty status changes among women between intervention and control groups were not significant during the follow-up.

**Table 4.** Changes in frailty status from baseline to 2 years in the intervention and control groups (percent with 95% confidence intervals).

Baseline frailty status	Post-intervention frailty status	Intervention group (%)^a^	Control group (%)^a^	Difference between groups^b^	95% CI for difference		*p*-Value for difference^c^
All study participants with baseline data, *n* = 1,138							
Non-frail (*n* = 794)	Non-frail	71.2	69.5	1.7	−4.8	8.2	0.613
	Pre-frail or frail	14.0	14.4	−0.4	−5.4	4.5	0.860
	No information	15.0	16.0	−1.1	−6.1	4.0	0.684
Pre-frail or frail (*n* = 344)	Non-frail	34.4	31.3	3.1	−7.6	13.9	0.568
	Pre-frail or frail	39.2	49.9	−10.7	−21.8	0.4	0.059
	No information	25.5	19.0	6.5	−2.5	15.6	0.158
Men with baseline data, *n* = 619							
Non-frail (*n* = 440)	Non-frail	71.1	71.9	−0.8	−9.5	7.8	0.854
	Pre-frail or frail	12.0	12.3	−0.3	−6.5	6.0	0.933
	No information	16.9	15.8	1.1	−5.9	8.1	0.763
Pre-frail or frail (*n* = 179)	Non-frail	45.7	30.0	15.7	1.1	30.4	0.036
	Pre-frail or frail	27.6	45.4	−17.8	−32.2	−3.5	0.015
	No information	26.7	24.6	2.1	−11.2	15.4	0.756
Women with baseline data, *n* = 519							
Non-frail (*n* = 354)	Non-frail	70.8	66.3	4.4	−5.4	14.2	0.376
	Pre-frail or frail	16.6	17.4	−0.8	−8.6	7.1	0.851
	No information	12.6	16.3	−3.7	−11.0	3.6	0.325
Pre-frail or frail (*n* = 165)	Non-frail	22.3	32.1	−9.9	−24.1	4.4	0.174
	Pre-frail or frail	54.0	54.5	−0.5	−16.4	15.5	0.953
	No information	23.7	13.3	10.3	−1.7	22.3	0.091

^a^ Estimated mean proportions from the model including interaction between baseline frailty status, sex and intervention group. Model adjusted for study site, education, and number of chronic diseases.

^b^ Estimated mean difference in proportions from the model including interaction between baseline frailty status, sex and intervention group. Model adjusted for study site, education, and number of chronic diseases.

^c^ *p*-Values for comparison of mean difference between the groups estimated from multinominal linear regression models.

No differences in changes in any frailty component separately were found between the intervention and control groups among all participants (results not shown). A beneficial effect of the intervention on physical activity level was, however, found among men. In the intervention group men reporting low level of physical activity at baseline became more often physically active during the intervention compared with the control group (difference between groups 30 percentage points, *p* = 0.038). Among women reporting weight loss at baseline, intervention group less often moved to the group without weight loss at 2 years (difference between groups 29 percentage points, *p* = 0.006). No other significant differences were seen for changes in frailty components.

## Discussion

This study found that a 2-year multidomain lifestyle intervention including nutritional counseling, physical exercise, cognitive training and social activity, and management of metabolic and vascular risk factors may be associated with beneficial effects in reducing the risk of frailty among older men. Among the components of frailty, the intervention had best effect on physical activity. Among women, such beneficial effect was not observed. The prevalence of pre-frailty or frailty at baseline was approximately 30% in this study population.

Previous studies targeting on outcomes such as frailty status, muscle strength or physical function among pre-frail or frail adults have shown evidence that multidomain interventions tend to be more effective compared with interventions focusing only on a single lifestyle-related factor [13]. Our study focusing on relatively healthy, community-dwelling older adults is one of the very few multidomain trials on the primary prevention of frailty. As frailty was not common among the participants at trial baseline, there was little room for improvement in frailty status, but we still observed that the intervention may have beneficial effects in terms of reversing frailty especially among older men. To the best of our knowledge, only one multidomain intervention study with long intervention period has found an association with development of frailty measured with Frailty Index [19]. The study population in that study was older and more frail compared with the current study.

We observed a sex difference in terms of benefits from the multidomain intervention in our study; men seemed to benefit more than women. Especially improvement was observed in physical activity among men. Sex differences may be partly explained by the level of baseline physical activity which was lower among men than women.

The main finding is thus that intervention was beneficial especially among men in increasing the physical activity level, which is one of the components of proposed by Fried. Benefits on physical activity and multidomain lifestyle interventions on persons who already have signs of frailty have been reported also earlier. A systematic review by de Labra et al. [36] showed that physical exercise interventions targeted to frail older people have several benefits across different outcome measurements, such as mobility, functional ability, balance, muscle strength and body composition The length of the interventions varied between six months to one year. In the study by Kim et al. [37] the definition of frailty was similar than in our study, involving the presence of three or more of the five frailty components from Fried’s criteria. This previous study showed that especially when 3-month exercise intervention was combined with nutritional supplement frailty reversal was seen in weight loss, exhaustion, low physical activity, and slow walking speed components, while only the change in muscle strength was not significant. However, the participants in this study were only women and older and more frail than in our study population. The contents and duration of the intervention and effect sizes have varied markedly among the studies, which makes it difficult to conclude what is the most effective way to prevent the onset of frailty or reverse the progression of frailty.

We also showed that among women, weight loss was more common in the intervention group after 2 years. This is not an unexpected consequence of a lifestyle intervention including diet and exercise, and in this context and population more likely a positive outcome than an indicator of frailty.

So far, the FINGER multidomain intervention has revealed beneficial effects on cognition [22], quality of life [38], daily functioning [23], and in this study we were also able to show that it may help in reversing frailty by especially increasing physical activity. As reported earlier, the intervention also resulted in no serious adverse events, and only minor events such as musculoskeletal pain were more frequently reported in the intervention group [22].

Although relatively modest, the results presented in this study might be clinically significant; it may be possible to slow down the progression of frailty via healthy lifestyle choices in persons who already have the first signs of frailty and who are physically inactive. Regular physical activity plays an important role in maintaining muscle strength, and is thus crucial for preventing sarcopenia, which is both biological foundation for frailty but also a mechanism contributing to consequences of frailty [39], However, as the most important individual factor of our result was found to be improvement in physical activity, the role of other intervention components remains unclear in our context.

Mortality risk among older people increases when moving from non-frail to physically frail within a few years [40]. Also, a decline in physical activity observed predicts premature mortality [41]. Therefore, regular assessments of the frailty status together with a support in maintaining physical activity are key elements when aiming at promoting health and functioning in older adults. Regarding the considerable consequences of frailty, it is important to try to postpone its onset instead of focusing on rehabilitation in people who already are frail.

This study has important strengths including the RCT design, a large sample size, a long intervention period, and carefully designed multidomain lifestyle intervention. However, we acknowledge several study limitations. First, the definition of frailty using Fried’s criteria is not very sensitive in a relatively well-functioning study population. Therefore, we were not able to detect small changes in frailty status and the present findings mostly indicate the intervention effect on physical activity. Second, a small number of pre-frail and frail individuals limited the statistical power of the analyses, and we were not able to analyze data for pre-frail and frail individuals separately. Third, the FINGER intervention was originally planned to study the effect of a multidomain intervention on cognitive functioning in older people at increased risk of cognitive decline. In addition, weight loss was assessed differently at baseline and at the follow-up, which may have introduced reporting bias. Furthermore, we did not ask whether weight loss was unintentional and therefore the weight loss component may have been confounded by intentional weight loss during the intervention. We also know that our study participants were relatively healthy, and their physical condition needed to be good to be able to safely participate in the exercise intervention. Therefore, participants with poorer health status were not included which limits the generalizability of the results. Therefore, it is obvious that additional RCTs are needed to investigate effects of lifestyle management on frailty phenotype in more detail.

## Conclusion

A multidomain lifestyle intervention targeting simultaneously multiple lifestyle and health domains may have potential to reverse frailty among older physically inactive men. The clinical significance holds promise of efficacy of lifestyle management through promoting physical activity for frailty prevention. Longer follow-up is needed to establish the effectiveness of multidomain lifestyle intervention on postponing the onset of frailty.

## Supplementary Material

Supplemental Material

## Data Availability

The datasets presented in this article are not readily available because public deposition of the de-identified dataset is not possible due to legal and ethical reasons, and complete deidentification is not possible as this investigation is part of an ongoing study. The study participants gave informed consent, which includes data use only under confidentiality agreement. Further, the data contain large amount of sensitive information and public data deposition may pose privacy concerns. Those fulfilling the requirements for viewing confidential data as required by the Finnish law and the Finnish Institute for Health and Welfare are able to access the data after completion of material transfer agreement. Requests to access the datasets should be directed to kirjaamo@thl.fi.
