# Supplementary material for: Effects of multidomain lifestyle intervention on frailty among older men and women – a secondary analysis of a randomized clinical trial
Source: Ann Med. 2025 Jan 1;57(1):2446699. doi: 10.1080/07853890.2024.2446699 (PMC11703011; doi:10.1080/07853890.2024.2446699)
Supplement: Supplemental Material [file IANN_A_2446699_SM1042.zip › Suppl_Mat/Ethical approval.pdf]

Koordinoiva eettinen toimikunta § 93 07.04.2009

94/13/03/00/09 UUSI TUTKIMUSSUUNNITELMA

94/13/03/00/2009

TMKE10 § 93

Esittelijä — Professori Leena Kivisaari

Julkisuus — Salassa pidettävä (JulkL 621/1999, 24 §)

Tutkimuksen nimi — FINNISH GERIATRIC INTERVENTION STUDY TO PREVENT COGNITIVE IMPAIRMENT AND DISABILITY (FINGER)

Kuvaus — Toimikunnan käsiteltäväksi on toimitettu uusi tutkimussuunnitelma.

Kyseessä on kansallinen lääketieteellinen monikeskustutkimus, jossa pyritään elintapoihin kohdistuvan intervention keinoin ehkäisemään muistitoimintojen heikkenemistä ja myöhentämään dementian ilmaantumista.

Tutkimuksesta vastaava henkilö
Dosentti Miia Kivipelto, THL

Tutkimuksen toimeksiantaja
Terveyden ja hyvinvoinnin laitos

Tutkimuksen rahoitus — Suomen Akatemia: SALVE-tutkimusohjelma

Toimitetut asiakirjat
Hakemus (lomake), 23.3.2009
Tutkimuksen suomenkielinen tiivistelmä, versio 2/20.3.2009
Liiteluettelo
Henkilörekisteriseloste, 1.3.2009
TVH:n eettinen arvio, 11.3.2009
Screening protocol, versio 2/16.3.2009
Kutsukirje 1, versio 1/19.3.2009
Tutkittavan tiedote ja suostumusasiakirja 1, versio 1/19.3.2009
Screening-käynnin lomake, versio 1/2.3.2009
CERAD-lomake, 23.3.2009
Sairauskyselylomake, versio 1/17.3.2009
Kutsukirje 2, versio 2/19.3.2009
Kutsukirje 3, versio 1/19.3.2009
Tutkittavan tiedote ja suostumusasiakirja 2, versio 1/19.3.2009
Tutkittavan tiedote ja suostumusasiakirja 3, versio 1/19.3.2009
Terveystietolomake, versio 1/20.3.2009
1. Baseline-käynnin lomake, versio 1/19.3.2009
Elämänlaatulomake, versio 2/20.3.2009
Saatekirje läheiselle, versio 1/17.3.2009
ADCS-ADL -lomake, 23.3.2009
Ruokapäiväkirja, versio 1/20.3.2009
Ruokavaliokysely, versio 1/20.3.2009
NTB-lomake, versio 1/22.3.2009
2. Baseline-käynnin lomake, versio 1/19.3.2009
DEPAI-lomake
DEPAQ-lomake, 31.3.2005

Kognitiivisen aktiivisuuden päiväkirja, versio 1/20.3.2009
Sosiaalisen aktiivisuuden päiväkirja, versio 1/20.3.2009
Intervention timetable, versio 1/19.3.2009
Curriculum Vitae

Päätösesitys

Eettinen toimikunta päättää

1. asiasta kokouskäsittelyssä,

2. periä lausuntomaksuna 0 euroa (STM:n asetus 1136/2008, 1 § 3 mom.).

Päätös

Toimikunta katsoo, että tutkimussuunnitelma ja sen liiteasiakirjat noudattavat lääketieteellisestä tutkimuksesta annetun lain (488/99 ja 295/04) ja asetuksen (986/99 ja 313/04) säännöksiä, tietosuojasäännöksiä sekä niitä lääketieteellistä tutkimusta ja tutkimuspotilaiden asemaa koskevia kansainvälisiä velvoitteita, joita ihmiseen kohdistuvalta lääketieteelliseltä tutkimukselta edellytetään.

Eettinen toimikunta pitää tutkimussuunnitelmaa eettisesti hyväksyttävänä ja päättää

1. antaa siitä puoltavan lausunnon. Toimikunta kuitenkin edellyttää, että asiakirjoihin tehdään seuraavat korjaukset:

- Tutkittaville tarkoitetuissa asiakirjoissa esimerkiksi kutsukirjeen ensimmäisessä lauseessa sana kognitiivinen tulee korvata jollakin helpommin ymmärrettävällä suomenkielisellä sanalla, esimerkiksi "ajatustoiminta".
- Jo ensimmäisessä tiedotteessa tulee kertoa, että tutkimuksessa kerätään tietoja myös eri rekistereistä, mihin tutkijat hankkivat asianmukaiset luvat.
- Magneettitutkimusta varten tarkoitetussa suostumusasiakirjassa tulee tutkittavilta varmistaa, ettei magneettitutkimukselle ole kontraindikaatiota.
- Tutkittavalta tulee kysyä, keneltä omaiseltaan hän haluaa kysyttävän tietoja ja saavan tietoa tutkittavasta.
- Tutkittavalta tulee myös pyytää suostumus omaisen mukana olemiseen ja tietojen saamiseen.
- Liiteenä 11 olevan kyselylomakkeen kysymykseen 50 nähden kysymyksissä 49 ja 51 esiintyy epäloogisuutta. Kysymykset tulee korjata johdonmukaisemmiksi. Lisäksi toimikunta pyytää vaihtaamaan liitteen 11 kysymyksessä 58 sanan "oppaatta" ilmaisuun "ilman opasta".
- Henkilörekisteriseloste tulee olla tieteellisen tutkimuksen henkilörekisteriselosteen lomakkeella. Selosteessa pitää myös kuvata, mitä tietoja tutkittavista ja heidän omaisiltaan kerätään.

Päätöstä koskeviin asiasisällöllisiin kysymyksiin vastaa tarvittaessa esittelijä.

2. periä käsittelymaksun esityksen mukaisesti.

Tutkimuksen saa aloittaa vasta sitten, kun puheenjohtaja on hyväksynyt tehdyt korjaukset.

Korjausten/selvitysten toimittaminen

Toimikunnan esittämiin korjauspyyntöihin tulee vastata kohta kohdalta. Tehtyjen muutosten tulee näkyä selkeästi muutetuissa asiakirjoissa. Lisäykset tekstiin tehdään tummennetulla vinotekstillä ja tekstin poistot joko yliviivaten tai harmaapohjalla. Tekstiin tehtyjä muutoksia, lisättyä ja poistettua tekstiä, ei saa merkitä sivujen marginaaliin esimerkiksi tekstinkäsittelyohjelman "näytä korjaukset" ("track changes") -toiminnon perusasetuksia käyttäen.

Liitteenä on toimitettava kopio pöytäkirjanotteesta, johon tehty korjaus/selvitys perustuu.

Koska kyse on hyvin pienistä korjauksista, niitä ei tarvitse toimittaa enää toimikuntaan käsiteltäväksi vaan ainoastaan toimikuntasihteerin kautta puheenjohtajan hyväksyttäväksi. Korjaukset pyydetään toimittamaan osoitteeseen: Heli Ådjers, Koordinoiva eettinen toimikunta, Biomedicum Helsinki 2 C, 7. krs, PL 705, 00029 HUS.

Lisätietoja

Esittelijä Leena Kivisaari p. 040 505 1006
Puheenjohtaja Katia Käyhkö p. 050 339 2688

Otteen tarkastamattomasta pöytäkirjasta oikeaksi todistaa

Helsingissä 14.4.2009

Heli Ådjers
sihteeri

Lähetetty tiedoksi 14.4.2009

Jakelu

Kivipelto Miia
Ahtiluoto Satu
